# High expression of RNA-binding motif protein 3 in esophageal and gastric adenocarcinoma correlates with intestinal metaplasia-associated tumours and independently predicts a reduced risk of recurrence and death

**DOI:** 10.1186/2050-7771-2-11

**Published:** 2014-06-17

**Authors:** Liv Jonsson, Charlotta Hedner, Alexander Gaber, Dejan Korkocic, Björn Nodin, Mathias Uhlén, Jakob Eberhard, Karin Jirström

**Affiliations:** 1 Department of Clinical Sciences, Division of Oncology-Pathology, Lund University, Skåne University Hospital, Lund 221 85, Sweden; 2 Science for Life Laboratory, AlbaNova University Center, Royal Institute of Technology, Stockholm 106 91, Sweden; 3 School of Biotechnology, AlbaNova University Center, Royal Institute of Technology, Stockholm 106 91, Sweden

**Keywords:** RBM3 expression, Prognosis, Esophageal and gastric adenocarcinoma, Intestinal metaplasia

## Abstract

**Background:**

High nuclear expression of the RNA-binding motif protein 3 (RBM3) has previously been found to correlate with favourable clinicopathological characteristics and a prolonged survival in several cancer forms. Here, we examined the clinicopathological correlates and prognostic significance of RBM3 expression in tumours from a consecutive cohort of upper gastrointestinal adenocarcinoma.

**Material and methods:**

Immunohistochemical RBM3 expression was analysed in tissue microarrays with primary radiotherapy- and chemotherapy-naive adenocarcinoma of the esophagus, gastroesophageal junction and stomach (n = 173). In addition paired samples of normal squamous epithelium (n = 53), gastric mucosa (n = 117), Barrett’s esophagus/gastric intestinal metaplasia (n = 61) and lymph node metastases (n = 71) were analysed. Kaplan-Meier analysis and Cox proportional hazards modelling was applied to assess the impact of RBM3 expression on overall survival (OS) and recurrence-free survival (RFS).

**Results:**

RBM3 expression was similar in primary tumours and lymph node metastases, but significantly higher in primary tumours and metastases arising in a background of intestinal metaplasia compared with cases without intestinal metaplasia (p < 0.001). RBM3 expression was significantly reduced in more advanced tumour stages (p = 0.006). Low RBM3 expression was significantly associated with a shorter OS in cases with radically resected (R0) tumours (HR 2.19, 95% CI 1.33-3.61, p = 0.002) and RFS in curatively treated patients with R0 resection/distant metastasis-free disease (HR = 3.21, 95% CI 1.64-6.30, p = 0.001). These associations remained significant in adjusted analysis (HR = 1.95, 95% CI 1.17-3.25, p = 0.010 for OS and HR = 3.02, 95% CI 1.45-6.29, p = 0.003 for RFS).

**Conclusion:**

High expression of RBM3 may signify a subset of upper gastrointestinal cancers arising in a background of intestinal metaplasia and independently predicts a reduced risk of recurrence and death in patients with these cancer forms. These findings are of potential clinical utility and merit further validation.

## Background

Esophageal cancer is the eighth most common cancer worldwide and the sixth most common cause of death from cancer [1], related to the high percentage of patients with unresectable or metastatic disease at diagnosis [2]. The incidence is increasing rapidly and in the past years a shift has occurred from squamous cell carcinoma to adenocarcinoma as the most common histological subtype in many Western countries [3,4]. The dominating risk factor for developing adenocarcinoma of the esophagus is gastro-esophageal reflux disease [5]. Adenocarcinoma of the stomach used to be the most common cancer globally, but the incidence has decreased in the past years and it is now the fourth most common cancer and the second leading cause of cancer death [1].

Both esophageal and stomach cancer often present with diffuse symptoms causing a delay in diagnosis, with the majority of cases being detected in more advanced disease stages leading to an impaired prognosis. Therefore, there is an urgent need to identify new prognostic and treatment predictive biomarkers for improved diagnostics and clinical management of patients with these cancer forms.

The RNA binding motif protein 3 (RBM3) has been identified as a promising prognostic and potentially treatment predictive biomarker in several major cancer forms e.g. breast, ovarian, prostate, bladder and colorectal cancer as well as malignant melanoma [6-11]. Specifically, while generally being upregulated in malignant compared to normal tissue, reduced expression of RBM3, in particular its nuclear location, has been demonstrated to be associated with an impaired prognosis [6-11]. Moreover, in epithelial ovarian cancer a link between reduced RBM3 levels and sensitivity to platinum based chemotherapy has been demonstrated *in vitro* and *in vivo*[6]. While the mechanistic basis for the association between high RBM3 expression and good prognosis needs to be further elucidated, some directions may be provided by the observed association between RBM3 expression and DNA integrity and repair [12], and the ability of RBM3 to attenuate stem-cell like properties of prostate cancer cells [13].

To our best knowledge, the prognostic significance of RBM3 in adenocarcinoma of the upper gastrointestinal tract has not yet been described. Therefore, in this study, the expression and prognostic impact of RBM3 was examined in a consecutive cohort of radiotherapy- and chemotherapy-naive adenocarcinoma of the esophagus and stomach (n = 173). In addition, RBM3 expression was analysed in a subset of matched normal squamous epithelium (n = 53), gastric mucosa (n = 117), intestinal metaplasia (IM) (Barrett’s esophagus or gastric IM, n = 72), and lymph node metastases (n = 71).

## Material and methods

### Study design and participants

The study cohort encompasses a consecutive cohort of 175 patients with esophageal and gastric adenocarcinomas who had been surgically treated in the University hospitals of Lund and Malmö from Jan 1st 2006 – Dec 31st 2010. The original and included cohort has been described in detail previously [14]. All tumours were histopathologically re-examined, including confirmation of diagnosis and number of lymph nodes with metastasis (re-classified following the standardized TNM 7 classification). Clinical data, information on recurrence, vital status and cause of death were obtained from the medical charts. The mean follow up time for patients alive was 5.2 years (range 2.7 – 7.7). Patient and tumour characteristics are summarized in Additional file 1.

Ethical permission was received from the regional ethical board of Lund University (ref nr 445/07).

### Tissue microarray construction

Tissue microarrays (TMAs) were constructed using a semi-automated arraying device (TMArrayer, Pathology Devices, Westminister, MD, USA). Duplicate tissue cores (1 mm) were obtained from viable, non-necrotic areas from all primary tumours. In addition, matched lymph node metastases were sampled from 81 cases, IM (gastric IM or Barrett’s esophagus) from 73 cases, normal squamous esophageal epithelium from 96 cases and normal gastric mucosa from 131 cases. Duplicate cores were obtained from different blocks of the primary tumour and different lymph node metastases in cases with more than one metastasis. Normal squamous epithelium and gastric mucosa was represented in single cores, and IM in 1–3 cores.

### Immunohistochemistry and staining evaluation

For immunohistochemical analysis, 4 μm TMA-sections were automatically pre-treated using the PT Link system and then stained in an Autostainer Plus (DAKO; Glostrup, Denmark) with the mouse monoclonal anti-RBM3 antibody AAb030038 (Atlas Antibodies AB, Stockholm, Sweden) diluted 1:1000. The specificity of the antibody has been validated previously [6,7].

The immunohistochemical staining was evaluated by two independent observers who were blinded to clinical and outcome data (LJ and AG). Scoring differences were discussed to reach consensus. For assessment of nuclear RBM3 expression, the estimated percentage of cells with nuclear RBM3 expression was recorded (NF), as well as the predominant nuclear intensity (NI) as 0 (negative), 1 (weak), 2 (moderate) and 3 (strong). A combined nuclear score (NS) was constructed by multiplying fraction and intensity. Cytoplasmic staining was not as evident, and recorded as 0 (absent) or 1 (present).

Immunohistochemical staining for Ki67 was performed in the Ventana BenchMark ULTRA system (Ventana Medical Systems, Tucson, AZ, USA) with a monoclonal antibody (clone MIB1, diluted 1:50, DAKO; Glostrup, Denmark). The staining was evaluated by two independent observers who were blinded to clinical and outcome data (CH and DK). The fraction of staining was categorized as follows: 0-1%, 2-10%, 11-20%, 20-50%, and >50%. For statistical analysis three categories were applied: 0-20%, 21-50% and >50%.

### Statistical analysis

Mann Whitney *U* test was applied for distribution analyses between the total nuclear RBM3 score and clinicopathological characteristics. Classification and regression tree (CRT) analysis [10,15] was used to assess the optimal prognostic cut-off for nuclear RBM3 expression in the primary tumours. Kaplan-Meier analysis and the log rank test was applied to estimate differences in overall survival (OS) and recurrence free survival (RFS) in strata according to high and low RBM3 expression. Cox regression proportional hazard’s modeling was used to estimate the impact of RBM3 expression on OS and RFS in both unadjusted and adjusted analysis, including age, sex, T-stage, N-stage, M-stage, differentiation and resection margins. Some subjects had no information on one or several markers and missing values were analysed separately. Missing values for categorical variables co-varied and the adjusted model did not converge due to many constant values. In order to avoid this, only patients with information on RBM3 expression were included in the unadjusted analysis. A backward conditional method was used for variable selection in the adjusted model. All test were two-sided. P-values <0.05 were considered significant. All statistical analyses were performed using IBM SPSS Statistics version 22.0 (SPSS Inc, Chicago, IL, USA).

## Results

### RBM3 expression in normal tissue, intestinal metaplasia, primary tumours and metastases

**Figure 1:**
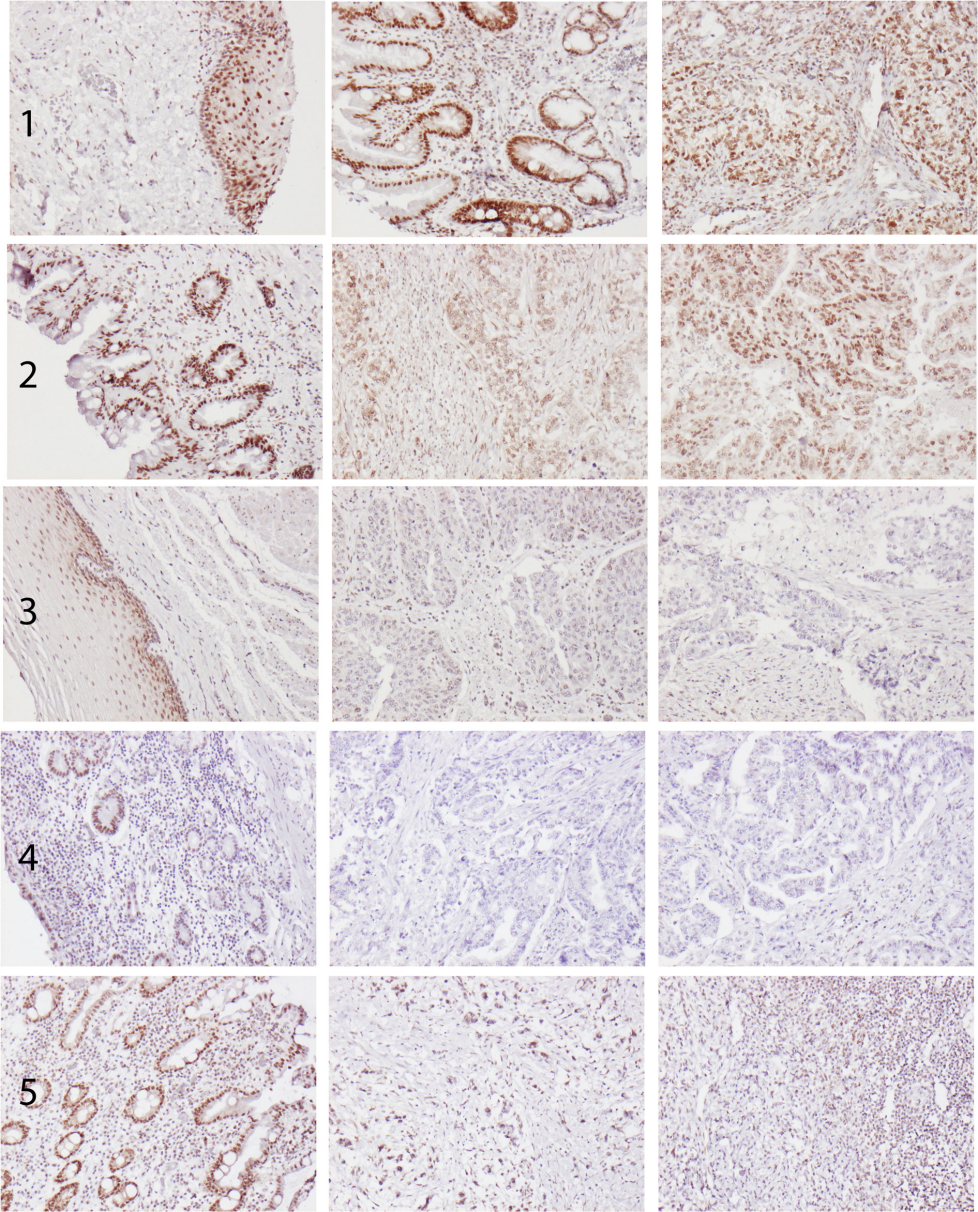
**Sample immunohistochemical images of RBM3 staining.** Images (10x magnification) of RBM3 expression in different tissue entities from five cases, from left to right: **(1)** Squamous epithelium, Barrett’s esophagus and primary tumour in a T1N0M1 esophageal cancer, **(2)** Barrett’s esophagus, primary tumour and lymph node metastasis in a T3N1M0 esophageal cancer, **(3)** squamous epithelium, primary tumour and lymph node metastasis in a T3N2M0 esophageal cancer, **(4)** normal gastric mucosa, primary tumour and lymph node metastasis in a T4N2M0 gastric cancer and **(5)** intestinal metaplasia, primary tumour and lymph node metastasis in a T3N1M0 gastric cancer with signet ring cell morphology.

**Figure 2:**
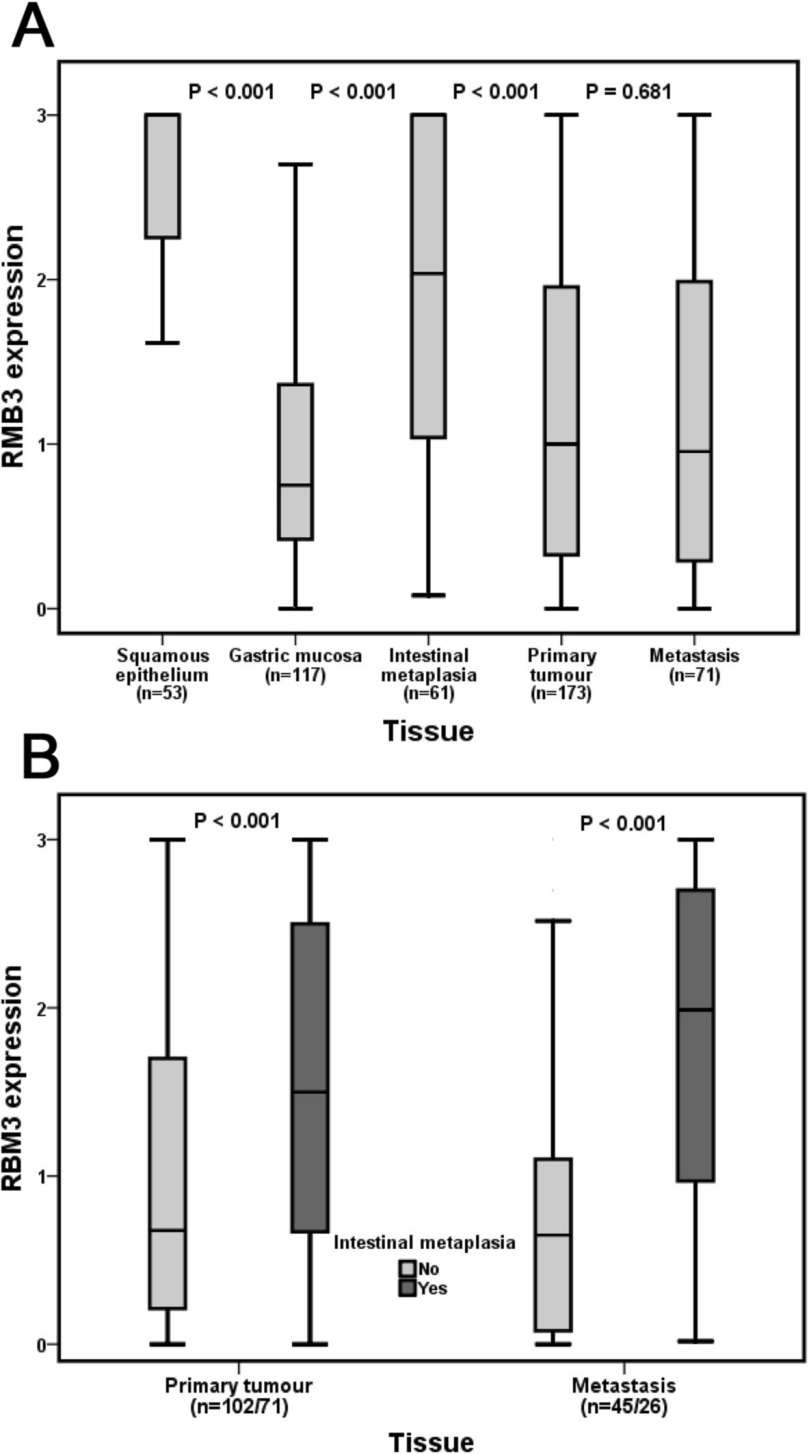
**RBM3 expression in normal tissue, intestinal metaplasia, primary tumours and metastases.** Box plots visualizing the distribution of RBM3 expression (nuclear score) in **(A)** squamous epithelium, gastric mucosa, intestinal metaplasia (Barrett’s esophagus or gastric intestinal metaplasia), primary tumours and metastases, and **(B)** primary tumours and metastases in cases with and without a background of intestinal metaplasia. The whiskers represent the smallest and the largest values, respectively (within 1.5 interquartile range).

RBM3 expression could be evaluated in 173/175 (98.9%) primary tumours and 71/75 (94.7%) metastases. There was no obvious heterogeneity between tissue cores. Sample immunohistochemical images are shown in Figure 1. As demonstrated in Figure 2A, RBM3 expression was significantly higher in normal-appearing squamous epithelium (n = 53) and IM (n = 61) compared to normal gastric mucosa (n = 117), primary tumours and metastases (p < 0.001), and RBM3 expression did not differ between primary tumours and metastases. Furthermore, RBM3 expression was significantly higher in primary tumours (p < 0.001) and metastases (p < 0.001) with a background of IM compared to cases without reported IM (Figure 2B). These significant associations were not altered in separate analysis of Barrett’s esophagus and gastric IM (data not shown). RBM3 expression in squamous epithelium and normal gastric mucosa did not differ significantly by the presence or absence of Barrett’s esophagus/IM (data not shown). In cases with IM RBM3 expression did not differ by the presence or absence of dysplasia, nor by the degree of dysplasia (data not shown).

### Associations of RBM3 expression in primary tumours with clinicopathological parameters

As demonstrated in Table 1 there was a significant association between reduced RBM3 expression and a more advanced T-stage (p = 0.006). There were no significant associations between RBM3 expression and any other clinicopathological parameters. Cytoplasmic RBM3 expression was not associated with any clinicopathological factors (data not shown). There was no significant correlation between expression of RBM3 and the previously investigated prognostic biomarker polymeric immunoglobulin receptor (PIGR) [14] (data not shown).

**Table 1:** Associations of RBM3 expression with clinicopathological parameters

	**RBM3 expression**	
	**Median (range)**	***p-value***
**Age**		
≤ average	1.00 (0.00-3.00)	0.212
>average	0.97 (0.00-3.00)	
**Gender**		
Female	0.85 (0.00-3.00)	0.100
Male	1.00 (0.00-3.00)	
**T-stage**		
T1	1.65 (0.00-3.00)	0.006
T2	1.49 (0.00-3.00)	
T3	0.98 (0.00-3.00)	
T4	0.40 (0.00-2.48)	
**N-stage**		
N0	1.02 (0.00-3.00)	0.568
N1	0.95 (0.00-3.00)	
N2	1.00 (0.00-3.00)	
N3	0.90 (0.00-3.00)	
**M-stage**		
M0	1.04 (0.00-3.00)	0.111
M1	0.61 (0.00-3.00)	
**Differentiation grade**		
High	0.91 (0.48-3.00)	0.732
Moderate	0.82 (0.00-3.00)	
Low	1.00 (0.00-3.00)	
**Resection margin**		
R0	1.00 (0.00-3.00)	0.948
R1	1.00 (0.00-3.00)	
R2	0.75 (0.00-3.00)	
**Location**		
Esophagus	1.14 (0.00-3.00)	0.158
Stomach	0.90 (0.00-3.00)	
**Growth pattern**		
Intestinal	1.00 (0.00-3.00)	0.379
Diffuse	0.94 (0.00-3.00)	
Mixed	1.55 (0.31-3.00)	

### Associations of RBM3 and Ki67 expression in primary tumours and metastases

**Figure 3:**
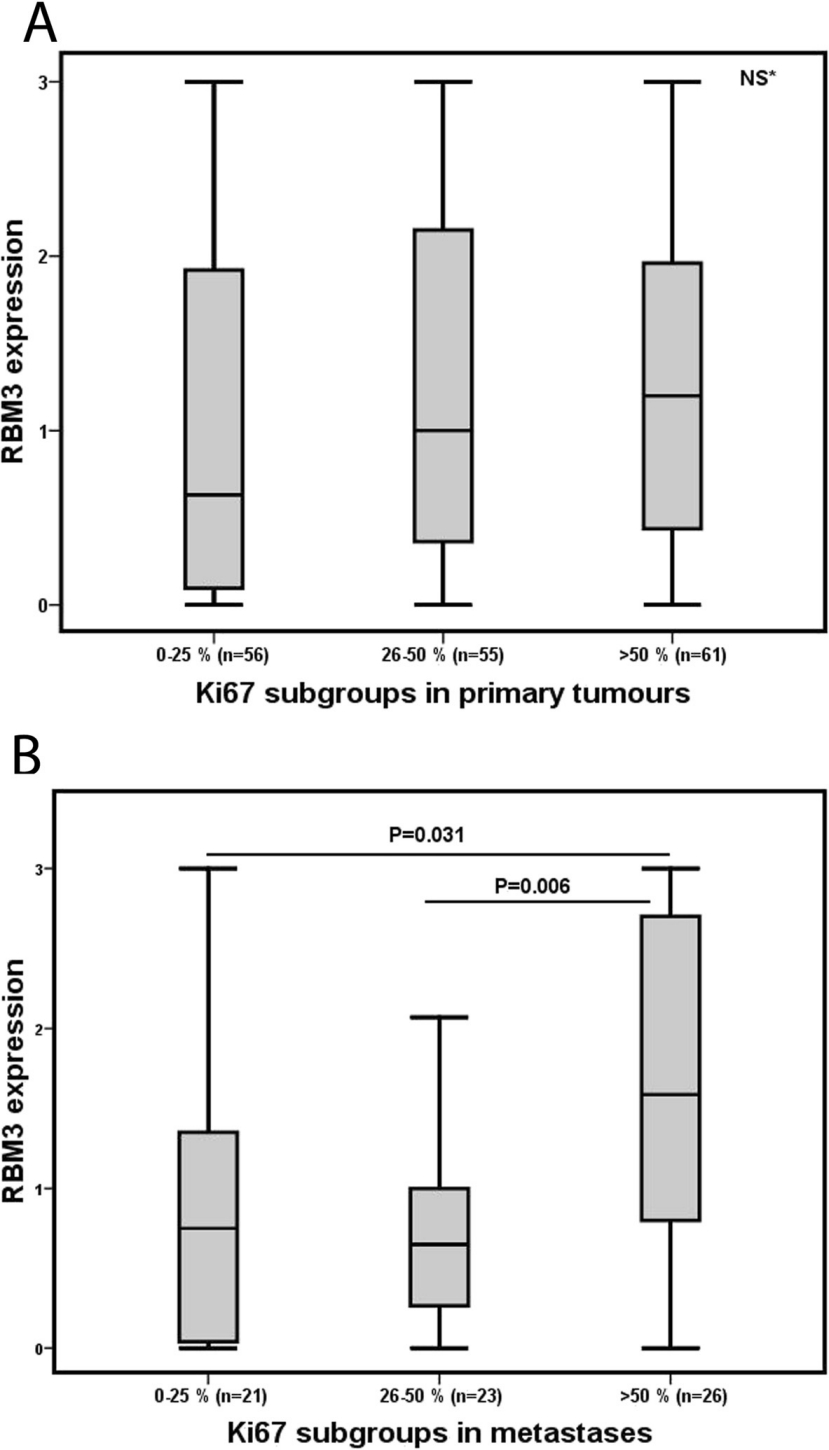
**RBM3 expression in relation to proliferation in primary tumours and metastases.** Box plots visualizing RBM3 expression (nuclear score) in relation to categories of Ki67 positivity in **(A)** primary tumours and **(B)** metastases. The whiskers represent the smallest and the largest values, respectively (within 1.5 interquartile range).

In primary tumours, there was no significant association between RBM3 expression and proliferation assessed by Ki67 expression (Figure 3A). In metastases, however, there was as positive correlation between high RBM3 expression and an increased proliferation (Figure 3B).

### Impact of RBM3 expression on survival

**Figure 4:**
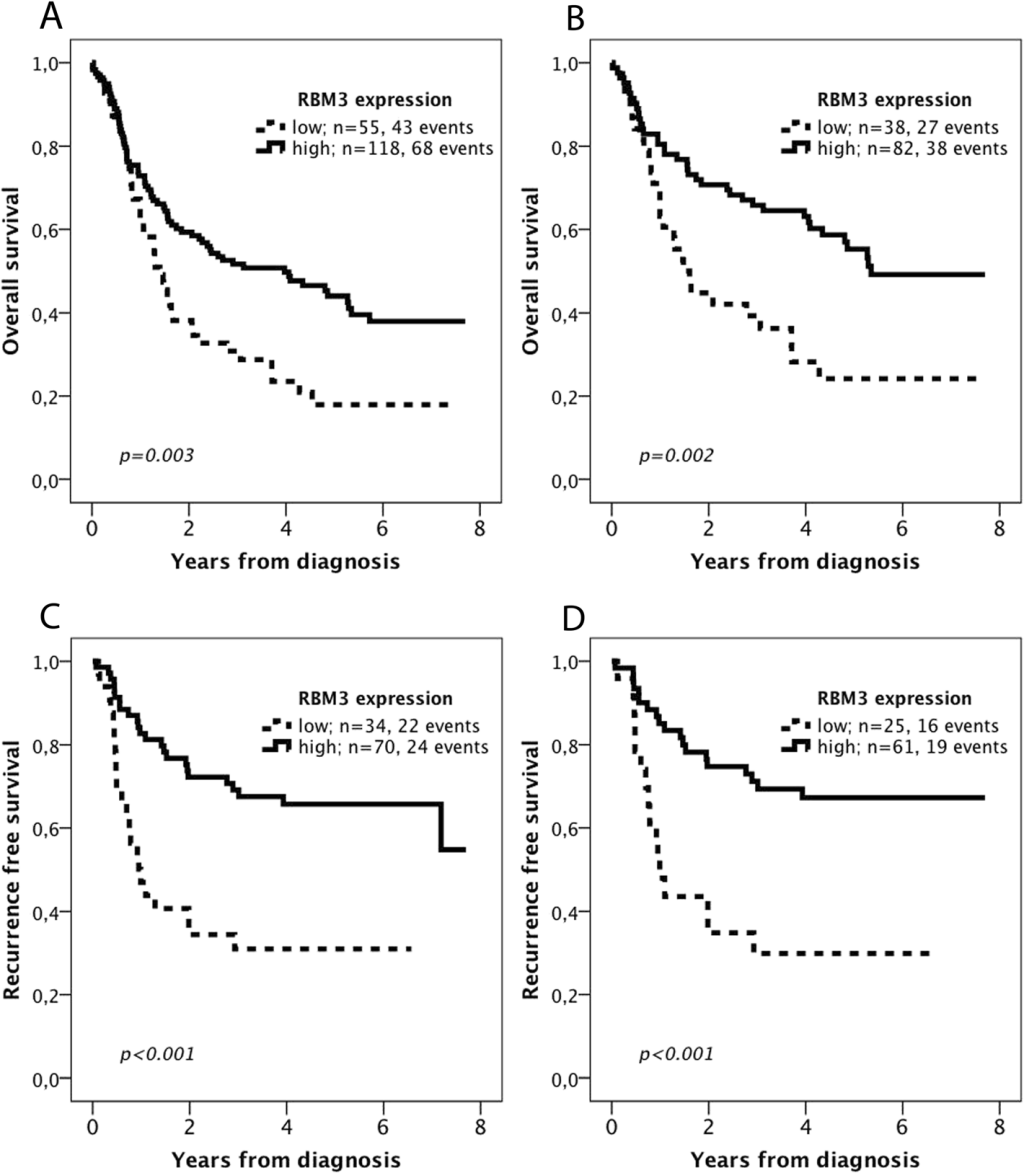
**Kaplan-Meier estimates of overall and recurrence free survival according to RBM3 expression.** Overall survival according to RBM3 expression in **(A)** the entire cohort, and in **(B)** patients with radically resected (R0) tumours. Recurrence free survival in **(C)** patients with R0 resection, and in **(D)** patients with distant-metastasis free (M0) disease and R0 resection.

According to the results of the CRT analysis a prognostic cut off at 0.452 was adopted for OS (low RBM3 expression < =0.452, high RBM3 expression >0.452) and at 0.440 for RFS (low RBM3 expression < =0.440, high RBM3 expression >0.440) (see Additional file 2). As demonstrated in Figure 4, low RBM3 expression was significantly associated with a reduced OS in the full cohort (p = 0.003, Figure 4A) and in cases with radically resected (R0) primary tumours (p = 0.002, Figure 4B). There was also a significant association between low RBM3 expression and a reduced RFS in cases with R0 resection (p < 0.001, Figure 4C) and in curatively treated patients with R0 resection and distant metastasis free (M0) disease (p < 0.001, Figure 4D).

As demonstrated in Table 2, the prognostic impact of RBM3 expression was confirmed in unadjusted Cox regression analysis; HR 2.19, 95% CI 1.33-3.61, p = 0.002 for OS in cases with R0 resection and HR = 3.21, 95% CI 1.64-6.30, p = 0.001 for RFS in curatively treated patients with R0 resection/distant metastasis-free disease. As further shown in Table 2, these associations remained significant in adjusted analysis (HR = 1.95, 95% CI 1.17-3.25, p = 0.010 for OS and HR = 3.02, 95% CI 1.45-6.29, p = 0.003 for RFS).

**Table 2:** Hazard ratios of death and recurrence according to clinicopathological factors and RBM3 expression in cases with radically resected primary tumours and radically resected primary tumours/distant metastasis-free disease, respectively

		**Overall survival - R0 resection**				**Recurrence free survival - R0 resection + M0 disease**				
		**Unadjusted**	**p-value**	**Adjusted**	**p-value**		**Unadjusted**	**p-value**	**Adjusted**	**p-value**
	**n (events)**	**HR (95% CI)**		**HR (95% CI)**		**n (events)**	**HR (95% CI)**		**HR (95% CI)**	
**Age**										
Continuous	120 (65)	1.04 (1.02-1.07)	<0.001	1.06 (1.03-1.09)	<0.001	86 (65)	1.00 (0.97-1.03)	0.909	1.03 (0.99-1.07)	0.100
**Gender**										
Female	26 (15)	1.00	0.843	1.00	0.915	16 (3)	1.00		1.00	
Male	94 (50)	0.85 (0.48.1.52)		0.97 (0.51-1.83)		70 (32)	2.83 (0.87.9.26)	0.084	3.31 (0.96-11.40))	0.058
**T-stage**										
T1	17 (5)	1.00		1.00		10 (1)	1.00		1.00	
T2	31 (17)	2.12 (0.81-6.00)	0.119	1.35 (0.46-3.99)	0.582	24 (7)	3.26 (0.40-6.00)	0.269	3.12 (0.30-31.88)	0.338
T3	56 (34)	2.59 (1.01-6.64)	0.047	1.04 (0.36-3.00)	0.942	43 (22)	6.42 (0.86-47.71)	0.069	3.90 (0.41-36.99)	0.236
T4	15 (9)	3.04 (1.01-9.08)	0.047	1.24 (0.35-4.35)	0.739	8 (5)	10.06 (1.17-86.42)	0.035	4.38 (0.35-55.05)	0.253
**N-stage**										
N0	45 (18)	1.00		1.00		35 (3)	1.00		1.00	
N1	23 (11)	1.30 (0.61-2.76)	0.489	1.67 (0.79-3.58)	0.188	18 (10)	8.08 (2.22-29.40)	0.001	12.36 (3.19-47.86)	<0.001
N2	27 (17)	1.98 (1.02-3.84)	0.045	2.65 (1.32-5.31)	0.006	22 (13)	10.53 (2.99-37.06)	<0.001	13.07 (3.52-48.53)	<0.001
N3	25 (19)	3.36 (1.75-6.47)	<0.001	4.85 (2.42-9.70)	<0.001	11 (9)	18.01 (4.81-67.44)	<0.001	27.19 (6.64-111.25)	<0.001
**M-stage**										
M0	96 (50)	1.00		1.00		96 (50)	1.00		1.00	
M1	10 (9)	2.74 (1.34-5.59)	0.006	1.55 (0.67-3.55)	0.304	10 (9)	-		-	
**Differentiation**										
High-Moderate	36 (19)	1.00		1.00		30 (9)	1.00		1.00	
Low	66 (40)	1.14 (0.66-1.97)	0.639	1.95 (0.78-4.91)	0.155	39 (20)	1.83 (0.83-4.03)	0.132	0.97 (0.33-2.88)	0.960
**RBM3 expression**										
High	82 (38)	1.00		1.00		61 (19)	1.00		1.00	
Low	38 (27)	2.19 (1.33-3.61)	0.002	1.95 (1.17-3.25)	0.010	25 (16)	3.21 (1.64-6.30)	0.001	3.02 (1.45-6.29)	0.003

Inclusion of tumour location (esophagus, gastroesophageal junction and stomach) in the multivariable model did not alter the independent prognostic significance of RBM3 expression and tumour location *per se* was not prognostic (data not shown).

Neither cytoplasmic RBM3 expression nor Ki67 expression were found to be prognostic (data not shown).

## Discussion

The results from this study demonstrate that reduced RBM3 expression is an independent factor of a shorter overall and recurrence free survival in patients with adenocarcinoma of the upper gastrointestinal tract. These findings add to the increasing number of cancer forms in which RBM3 expression has proven to be an independent prognostic and treatment predictive biomarker [6-12]. In line with previous studies, RBM3 was mainly expressed in the nucleus, and its prognostic value evident for nuclear but not cytoplasmic expression.

In the here analysed consecutive cohort, all cases receiving neoadjuvant chemotherapy had been excluded and only a minor proportion, 9.6%, had received adjuvant treatment. Thus, the association of RBM3 expression with a favourable prognosis is not likely explained by a treatment predictive effect. Nevertheless, given that RBM3 expression has previously been shown to correlate with sensitivity to cisplatin treatment in epithelial ovarian cancer cells [6], it would also be of interest to examine the potential treatment predictive effect of RBM3 in upper gastrointestinal adenocarcinoma in future studies. Neoadjuvant chemotherapy, nowadays commonly including oxaliplatin [16], has become mainstay in the treatment of adenocarcinomas of the stomach [17] however, for esophageal cancers studies show contradictive results regarding neoadjuvant therapy [18,19]. Thus, the utility of RBM3 as a predictive marker for response to neoadjuvant chemotherapy merits further study, preferably in tumour samples from patients enrolled in randomized treatment trials.

In general, RBM3 expression seems to be upregulated in preinvasive and cancerous tissues compared to their normal counterparts [10,20,21]. In the present study, RBM3 expression was found to be higher in benign-appearing squamous epithelium and intestinal metaplasia (Barrett’s esophagus or gastric intestinal metaplasia) compared to primary and metastatic cancer, whereas the expression in normal gastric mucosa was significantly lower than in all other tissue entities.

Of note, RBM3 expression was significantly higher in tumours with associated Barrett’s esophagus or intestinal metaplasia compared to tumours without the presence of these pre-neoplastic lesions. These findings are of potential interest as they implicate a role for RBM3 in the intestinal pathway of carcinogenesis of upper gastrointestinal adenocarcinoma. Given that high RBM3 expression was demonstrated to be an independent favourable prognostic factor, its association with intestinal-type epithelium is also in line with findings from a previous comprehensive analysis of adenocarcinoma of the distal esophagus and esophagogastric junction, in which cases with associated intestinal-type mucosa were demonstrated to exhibit more favourable histopathological characteristics and a significantly prolonged survival compared to cases with associated cardiac-type mucosa [22].

In contrast to our previous study on malignant melanoma, where RBM3 was found to be downregulated in metastases compared to primary tumours [9], RBM3 expression was similar in primary tumours and matched lymph node metastases. Apart from indicating that loss of RBM3 is an early event in adenocarcinoma of the upper gastrointestinal tract, these findings also support that analysis of RBM3 in the primary tumour is sufficient for prognostic and, potentially, treatment predictive purposes.

In the present study, we also examined the associations of RBM3 expression with the proliferation marker Ki67 in primary tumours and metastases. While no correlation was found in primary tumours, there was a significant association between higher RBM3 expression and proliferation in metastases. The relevance of this finding, which may well be coincidental, is however less evident, as neither RBM3 expression in the metastases nor Ki67 expression in primary tumours or metastases were significantly associated with clinical outcome in the here analysed cohort. Some studies have demonstrated an association between RBM3 expression and proliferation *in vitro* as well as in various normal proliferating and malignant tissues [21], however in our previous study on malignant melanoma, there was no significant association between expression of RBM3 and Ki67, but a significant inverse correlation between RBM3 expression and another proliferation marker, the minichromosome maintenance 3 (MCM3) protein [23]. Moreover, in that study, Ki67 expression was found to be prognostic, however not independent of other factors, while MCM3 was an independent factor of poor prognosis [23,24].

A limitation to the present study is the retrospective setting, where curative intent may be difficult to determine. However, loss of RBM3 expression was found to be prognostic in all cases with radically resected primary tumours, with our without inclusion of the small number of cases having distant metastases. The reasons for surgical removal of the primary tumour in cases with R0 resection/distant metastasis were either bleeding of the primary tumour or presence of non-locoregional lymph node metastases and, of note, RBM3 remained an independent factor also after adjustment for distant metastasis in this category.

Another possible limitation is the potential sampling bias and heterogeneity issue associated with use of the TMA technique. It should however be pointed out that these issues will remain even after analysis of full-face sections, as these also represent only a part of the tumour. Moreover, immunohistochemical analysis of multiple sections is not a feasible approach in biomarker studies. Therefore, a relative strength of the TMA design used in the present study is that duplicate cores were, in the majority of cases, obtained from different blocks of the primary tumour and different lymph node metastases in cases with more than one metastasis. Moreover, TMA-based biomarker analyses have been demonstrated to give equal or even improved prognostic information compared to analysis of full-face sections [25].

In conclusion, the results from this study demonstrate that reduced RBM3 expression is an independent factor of poor prognosis in patients with adenocarcinoma of the upper gastrointestinal tract. Moreover, RBM3 expression was found to be significantly higher in tumours with associated Barrett’s esophagus or gastric intestinal metaplasia compared to tumours without the presence of these pre-neoplastic lesions. These findings are of potential interest as they implicate a role of RBM3 in different pathways of the pathogenesis of adenocarcinomas of the esophagus, esophagogastric junction and stomach. The value of RBM3 for prognostication and treatment stratification in patients with upper gastrointestinal adenocarcinoma should be pursued in future studies.

## Abbreviations

RBM3: RNA-binding motif protein 3; OS: Overall survival; RFS: Recurrence free survival; R0: Radical resection; HR: Hazard ratio; CI: Confidence intervall; TMA: Tissue micro array; NF: Nuclear fraction; NI: Nuclear intensity; NS: Nuclear score; CRT: Classification and regression tree; IM: Intestinal metaplasia; MCM3: Minichromosome maintenance 3.

## Competing interests

The authors declare that no competing interests exist.

## Authors’ contributions

LJ evaluated the immunohistochemical stainings, performed the statistical analyses and drafted the manuscript. CH collected and re-examined clinicopathological data and assisted with TMA construction. AG evaluated the immunohistochemical stainings and assisted with the statistical analysis. DC evaluated the immunohistochemical stainings. BN constructed the tissue micro array and performed the IHC stainings. MU contributed with antibody validation. JE assisted with collection of clinical data. KJ conceived of the study, evaluated the immunohistochemistry, and helped draft the manuscript. All authors read and approved the final manuscript.

## Supplementary Material

Additional file 1 Patient and tumour characteristics for the entire cohort, esophagus, cardia and stomach. Click here for file

Additional file 2 Classification regression tree analysis for selection of prognostic cutoffs regarding RBM3 expression in (A) OS in the entire cohort and (B) RFS in patients with R0 resection. Click here for file
