# Supplementary figures and images for: High expression of RNA-binding motif protein 3 in esophageal and gastric adenocarcinoma correlates with intestinal metaplasia-associated tumours and independently predicts a reduced risk of recurrence and death
Source: Biomark Res. 2014 Jun 17;2:11. doi: 10.1186/2050-7771-2-11 (PMC4067631; doi:10.1186/2050-7771-2-11)

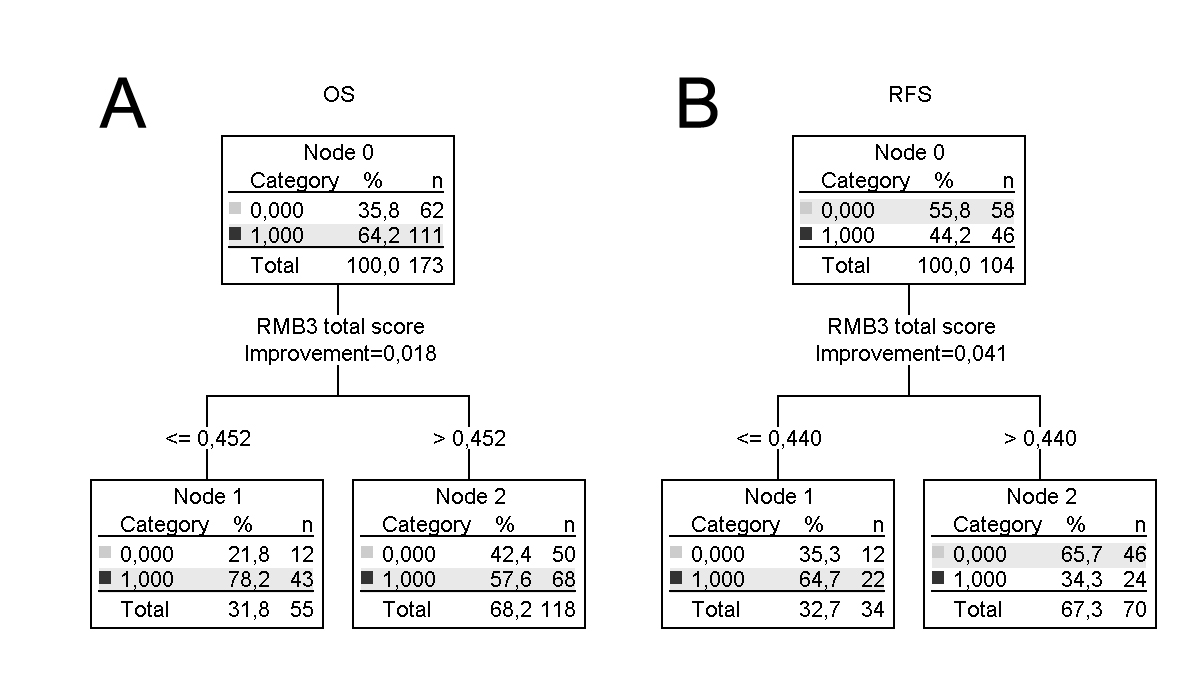


Additional file 2

Supplement: Additional file 2 — Classification regression tree analysis for selection of prognostic cutoffs regarding RBM3 expression in (A) OS in the entire cohort and (B) RFS in patients with R0 resection. [file 2050-7771-2-11-S2.docx]
